# Supplementary material for: Inflammatory factors and risk of lung adenocarcinoma: a Mendelian randomization study mediated by blood metabolites
Source: Front Endocrinol (Lausanne). 2024 Aug 27;15:1446863. doi: 10.3389/fendo.2024.1446863 (PMC11384989; doi:10.3389/fendo.2024.1446863)
Supplement: Supplementary file 1 [file DataSheet1.zip › supplementary files/S2/GCST90200097/forest.pdf]

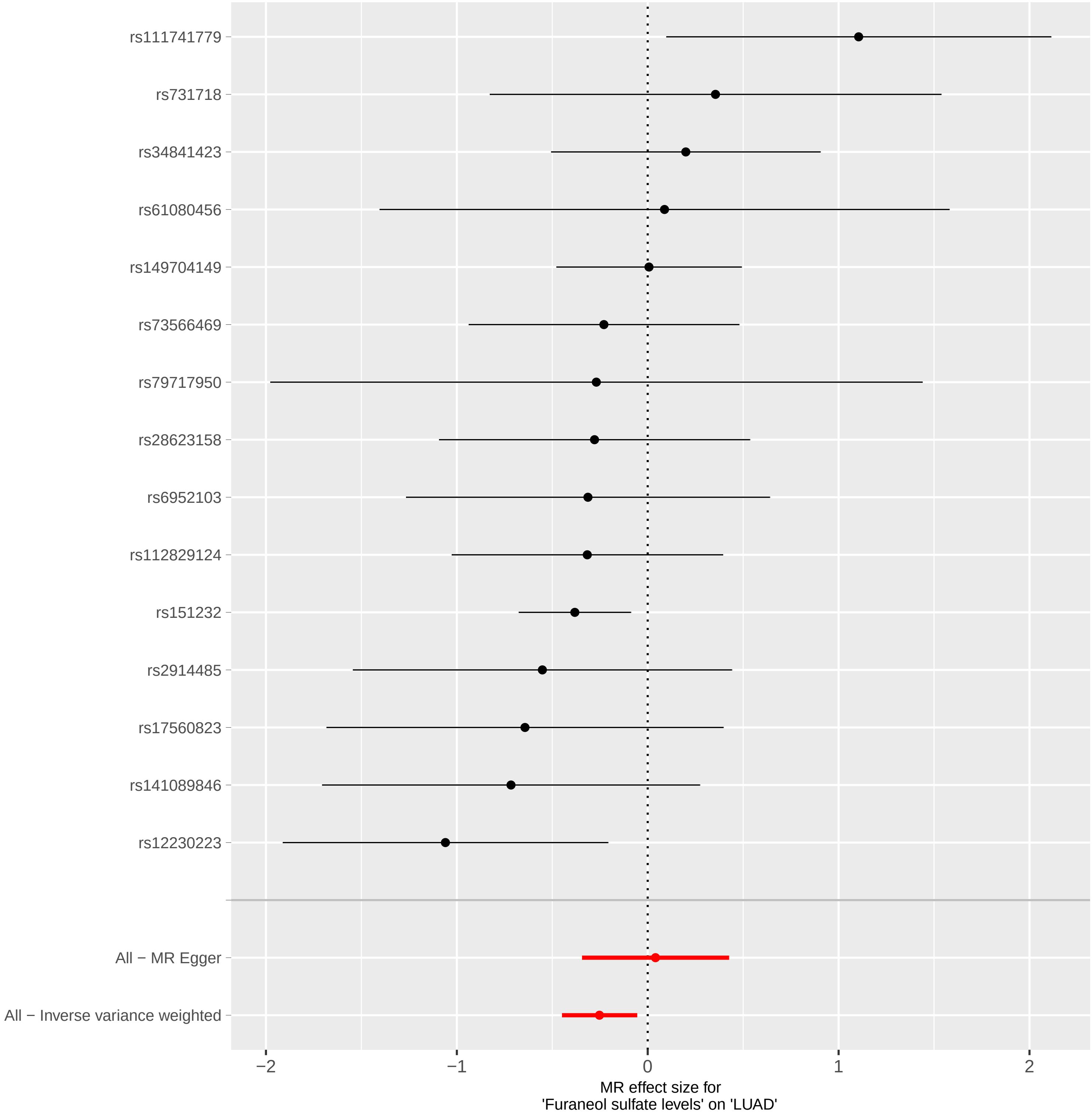
rs111741779
rs731718
rs34841423
rs61080456
rs149704149
rs73566469
rs79717950
rs28623158
rs6952103
rs112829124
rs151232
rs2914485
rs17560823
rs141089846
rs12230223
All − MR Egger
All − Inverse variance weighted
−2
−1
0
1
2
MR effect size for
'Furaneol sulfate levels' on 'LUAD'
